# Organoid culture media formulated with growth factors of defined cellular activity

**DOI:** 10.1038/s41598-019-42604-0

**Published:** 2019-04-17

**Authors:** Manuela Urbischek, Helena Rannikmae, Thomas Foets, Katharina Ravn, Marko Hyvönen, Marc de la Roche

**Affiliations:** Department of Biochemistry, University of Cambridge, Cambridge, United Kingdom

**Keywords:** Biological models, Cell biology

## Abstract

The media formulations necessary for deriving and sustaining organoids from epithelial tissues such as prostate, colon, gastric, liver, pancreas, and others have been established. Critical components of organoid media are a set of growth factors that include R-spondins and BMP signalling antagonists such as Noggin or Gremlin 1. Currently, the practical limitations for formulating organoid media of reproducible potency and larger-scale media production that have hampered further technological applications of organoid technology include: the cost of growth factors such as R-spondins and Gremlin 1/Noggin and their production as defined specific activities free of contaminants that may affect organoid growth. Here we report the production of highly pure recombinant Gremlin 1 and R-spondin 1 from bacterial expression for use in organoid media. We detail the workflow for Gremlin 1 and R-spondin 1 expression, purification, quantification of cellular activity, quality control and use in media formulated for culturing organoids derived from a number of tissues. The development of precisely formulated, cost-effective media of defined specific activity will engender the development of novel applications for organoid technology.

## Introduction

The development of organoid technology has proven to be scientifically transformative with tremendous potential for applications in the fields of epithelial and cell biology and pathology and for clinical applications such as the generation of patient-derived organoid biobanks^1,2^. The critical component of organoid technology is the formulation of culture media with essential growth factors, in particular - R-spondins, which potentiates Wnt pathway activity in epithelial stem cells and Noggin or Gremlin 1 to inhibit differentiation cues from the BMP pathway. Currently, the production of growth factors relies on eukaryotic expression systems to ensure correct disulphide bond formation and macromolecular folding. Typically, in eukaryotic expression systems, growth factors are secreted into the cell culture media from the expression hosts, and this conditioned media containing the growth factor of choice (and all other proteins these cells naturally secrete) is then diluted directly into organoid culture media. However, R-spondin 1 and Gremlin 1/Noggin expressing cell lines are not widely available and the use of conditioned media presents the problems of batch-to-batch variation of growth factor activity. Moreover, the presence of proteins, including growth factors from serum present in cell culture media as well as those secreted from those cells, may affect organoid growth. Commercially available purified growth factors can also be used to supplement organoid media however, these are expensive for medium to large scale applications such as genetic and chemical library screening or clinical biobanking. Moreover, commercial growth factors preparations may retain impurities such as media serum components preventing accurate and reproducible determination of their cellular activities.

Here, we report a protocol for the generation of highly pure recombinant Gremlin 1 and R-spondin 1 from bacterial expression with negligible levels of endotoxin and free from impurities endemic to production using eukaryotic expression systems. We detail protocols for the determination of the cellular activities of the growth factors enabling the generation of batches of media of reproducible potencies for organoids culture circumventing the issue of batch-to-batch variation. Our workflow for generating media of defined and reproducible potencies has broad application for culturing organoids derived from a number of tissue types.

## Results

**Figure 1:**
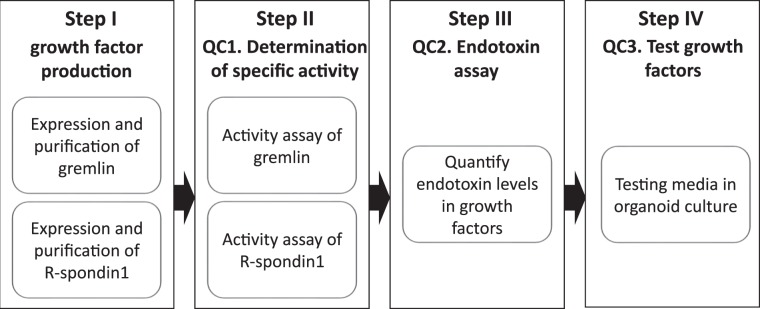
Overview of the procedure. The generation of recombinant R-spondin 1 and Gremlin and subsequent characterisation steps for use in organoid media are as follows: Step I - Recombinant growth factors R-spondin 1 and Gremlin 1 are expressed in bacteria and purified. Step II – Cellular activities of Gremlin 1 and R-spondin 1 were determined from corresponding reporter assays. Step III – A quality control step is used to ensure minimum endotoxin levels (from growth factor expressing bacteria) are present in the preparations using the limulus amoeboid lysate (LAL) assay. Step IV - media formulated with defined activities of R-spondin 1 and Gremlin 1 is tested for the ability to support growth of murine intestinal epithelial organoids. We have further outlined abridged purification protocols for Gremlin 1 and R-spondin 1 in Materials and Methods, ideally suited to laboratories without access to production-scale chromatography equipment, for production of MBP-R-spondin 1 and Gremlin 1 of sufficient purity and cellular activity for organoid culture.

The R-spondin family and the Noggin/Gremlin 1 BMP inhibitors are essential growth factors for culturing organoids derived from a number of epithelial tissues. We report here a key refinement in the culture of organoids – the use of highly pure preparations of R-spondin 1 and Gremlin 1 of defined cellular activities derived from bacterial expression. We detail the workflow for growth factor production, quality control and formulation of organoid culture media (Fig. 1) as follows:

### Production of R-spondin 1

**Figure 2:**
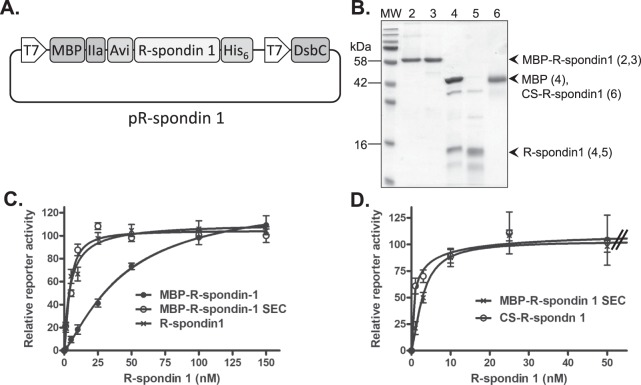
Bacterial expression and production of R-spondin 1. (**A**) Format of the pR-spondin 1 expression vector based on the pETDuet backbone. pR-spondin 1 is a modification of a previously R-spondin 1 expression vector^10^. (**B**) SDS-PAGE analysis of fractions from generation of R-spondin 1. For each fraction, 2 μg of protein was loaded. Lane numbers (top and in brackets) are: 2 – MBP-R-spondin 1 before SEC, 3 - MBP-R-spondin 1 after SEC, 4 – products of Thrombin cleavage, 5- final R-spondin 1 preparation and 6 – CS-R-spondin 1. MW, molecular weight standards. (**C**) Cellular activity of R-spondin 1 fractions measured as WPC50s: *circles* - MBP-R-spondin 1 before SEC, WPC50 = 45 ± 9.9 nM, *diamonds* - MBP-R-spondin 1 after SEC, WPC50 = 4.0 ± 0.53 nM and *squares* - final R-spondin 1 preparation, WPC50 = 4.5 ± 0.80 nM. (**D**) Cellular activities of MBP-R-spondin 1 and CS-R-spondin 1 measured as WPC50s: *circles* - MBP-R-spondin 1 after SEC, *squares* - CS-R-spondin 1, WPC50 = 1.2 ± 0.69 nM. To delineate between the MBP-R-spondin 1 and CS-R-spondin 1 dose-response data sets, we have limited presentation of the results to protein concentrations up to 50 nM.

The expression vector for R-spondin 1 (Fig. 2A) has been modified from a previous report^3^ to include the coding sequence for the 9 amino acid Avi-tag fused to the N-terminus of the R-spondin 1 that confers enhanced solubility and specific activity of the expressed protein.

R-spondin activity is particularly sensitive to the correct configuration of disulphide linkages. In addition to the R-spondin 1 expression cassette, pR-spondin 1 drives expression of *DsbC*, a disulphide isomerase that promotes the correct configuration of disulphide linkages. We express R-spondin 1 in the NEB Shuffle T7 *E. coli* strain that also expresses additional *DsbC* in the cytoplasm. Moreover, following lysis of expressing bacteria and batch purification by Nickel-NTA agarose we subject the expressed R-spondin 1 to an *in vitro* disulphide shuffling step in the presence of reduced and oxidised glutathione. Nonetheless, a subsequent SEC step removes approximately 60% of the inactive R-spondin 1 protein aggregates found in the void volume (Suppl. Fig. 1). Non-aggregated MBP-R-spondin 1 resolves in fractions corresponding to the correct molecular mass of 58 kDa and is an essentially pure preparation demonstrated by migration as a single band of protein by SDS-PAGE (Fig. 2B; Suppl. Figs 1, 2). Cellular activities of the MBP-R-spondin 1 fractions, measured as WPC50s, increase greater than 10-fold with SEC, from 45 ± 9.9 nM to 4.0 ± 0.53 nM (Fig. 2C; Table 1). The purified MBP-R-spondin can be used to supplement organoid media at a concentration of 25 nM.

**Table 1:** Cellular activities and endotoxin contamination in R-spondin 1 and Gremlin 1 fractions.

Protein	Cellular activity* (nM)	Endotoxin levels (EU/mg protein)	Endotoxin levels in organoid media EU/ml	Estimated cost per litre of organoid media (£)**
MBP-R-spondin 1 before SEC	45 ± 9.9	1.01	0.001	n/a
MBP-R-spondin 1 after SEC	4.0 ± 0.53	4.22	0.005	<10
R-spondin 1	4.5 ± 0.80	17.41	0.026	<10
CS-R-spondin 1	1.2 ± 0.69	9.18	0.005	>5,000
Gremlin 1	6.4 ± 0.65	6.66	0.002	<10
CS-Gremlin 1	6.0 ± 0.33	not detected	not detected	>3,500

*Cellular activities for R-spondin 1 are calculated as WPC50 and for Gremlin 1 are calculated as IC_50_ for BMP2-induced ALP. **Cost of materials.

Removal of the MBP moiety by Thrombin cleavage followed by cation exchange chromatography produces pure R-spondin 1 with a WPC50 of 4.5 ± 0.80 nM, similar to MBP-R-spondin 1 after SEC (Fig. 2C; Table 1). R-spondin 1 migrates on SDS-PAGE gels as a single band at the expected molecular mass of 16 kDa (Fig. 2B). Commercially-sourced R-spondin 1 (CS-R-spondin 1) retains 118 amino acids at the C-terminus not present in bacterially-derived R-spondin 1 and migrates as a 42 kDa protein instead of the predicted 26 kDa, presumably due to glycosylation (Fig. 2B). The WPC50 of CS-R-spondin 1 was determined to be 1.2 ± 0.69 nM, based on the assumption of a single protein species with a molecular mass of 42 kDa (Fig. 2D; Table 1). A typical yield of purified MBP-R-spondin 1 and R-spondin 1 is approximately 2.5 mg and 1 mg of protein per L of Luria broth. For organoid culture, 2.5 mg or 1 mg of the respective proteins is sufficient to supplement approximately 3 L of organoid media at a concentration of 25 nM (Table 2). Typical levels of contaminating endotoxin in the final MBP-R-spondin 1 and R-spondin 1 preparations are similar to CS-R-spondin 1 and >20-fold less than the LOCE threshold value of 0.5 EU/ml endotoxin when diluted into organoid culture media (Table 1).

**Table 2:** Media components for the culture of various organoid types.

Component	Source of derived organoids				
	murine small intestinal epithelial	murine colon epithelial	murine mammary gland	Human colon epithelia	Human colon carcinoma
Base media*	1×	1×	1×	1×	1×
B27 suppl.	1×	1×	1×	1×	1×
N2 suppl.	1×	1×	1×	1×	1×
NAC	1.25 mM		—	1 mM	1 mM
EGF	50 μg/ml	50 μg/ml	—	50 μg/ml	50 μg/ml
R-spondin 1	25 nM	25 nM	25 nM	25 nM	25 nM
Gremlin 1	25 nM	25 nM	25 nM	25 nM	25 nM
WCM	—	0.5×	—	—	0.5×
NRG-1	—	—	100 ng.ml	—	—
A83-01	—	—	—	500 nM	500 nM
SB202190	—	—	—	10 μM	10 μM
PGE2	—	—	—	10 mM	10 mM
FGF				100 μg/ml	100 μg/ml
Gastrin I	—	—	—	10 nM	10 nM
Nicotinamide	—	—	—	10 mM	10 mM
Primocin	—	—	—	1×	1×

Organoids derived from murine small intestinal epithelial, colon epithelial, mammary gland and human colon epithelia and patient-matched colon carcinoma were formulated as previously described^5,15,16^ and include optimised concentrations of bacterially-derived R-spondin 1 and Gremlin 1. ^*^Base media is DMEM/F12 media containing 2 mM glutamine, 10 mM HEPES and 0.5 U/ml Penicillin/streptomycin.

For the production of MBP-R-spondin 1 from 2 L of Luria broth we estimate a time commitment of two days and material cost of <£10 per litre of organoid media (Table 1). In contrast, the cost of CS-R-spondin 1 for one litre of organoid media is >£5,000 per litre.

### Production of Gremlin 1

**Figure 3:**
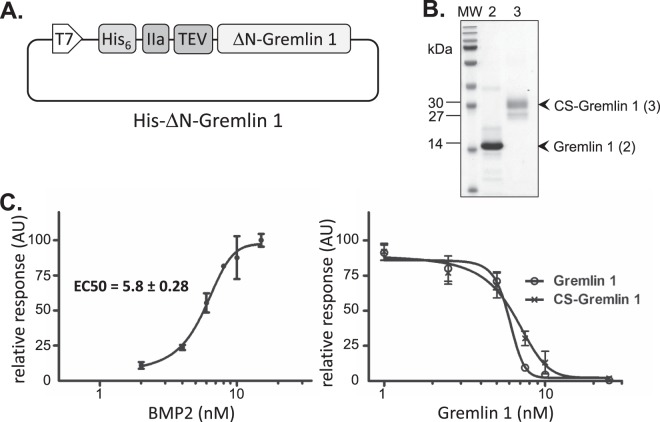
Bacterial expression and production of Gremlin 1. (**A**) Format of the His-ΔN-Gremlin 1 plasmid for production of Gremlin 1^4^. (**B**) SDS-PAGE of purified Gremlin 1 (lane 2) and CS-Gremlin 1 (lane 3). For each protein, 2 μg was loaded. MW, molecular weight standards. (**C**) Activity assay for purified Gremlin 1 based on its inhibition of BMP2-induced ALP activity in C2C12 cells. *Left graph* – Determination of BMP2 activity to establish a suitable concentration for use in Gremlin 1 assays. *Right graph*: Inhibition of ALP activity, induced with 8 nM BMP2 treatment, by Gremlin 1 and CS-Gremlin 1. IC_50_ values for Gremlin 1 and CS-Gremlin were determined as 6.4 ± 0.65 nM and 6.0 ± 0.33 nM respectively.

The vector for bacterial expression of recombinant human Gremlin 1 (His-ΔN-Gremlin 1; Fig. 3A) and the purification procedure has previously been described^4^. Bacterially-expressed recombinant Gremlin 1 associates with inclusion bodies requiring suspension of the protein under denaturing conditions and a refolding/disulphide shuffling step for generation of natively-folded protein. Subsequent anion exchange chromatography leads to an essentially pure preparation of Gremlin 1 that can be used in organoid media formulations at a concentration of 25 nM.

A final reverse phase chromatography step resolves a highly pure fraction of Gremlin 1 shown by a single band on SDS-PAGE migrating at the expected molecular mass of 14 kDa (Fig. 3B). By contrast, the 30 kDa commercially-sourced Gremlin 1 (CS-Gremlin 1) protein, consisting of amino acids 25–284, migrates as two bands on SDS-PAGE gels, at 27 kDa and 30 kDa (Fig. 3B). The reduced mobility and diffuseness of CS-Gremlin 1 migration is typical of glycosylated proteins derived from eukaryotic expression systems. We calculated CS-Gremlin 1 cellular activity assuming a single protein species with a molecular mass of 30 kDa.

The cellular activity of bacterially-derived Gremlin 1 is measured as the IC_50_ for the inhibition of BMP2-induced ALP activity in C2C12 cells. We first assayed BMP2 ALP activity and determined an EC_50_ value of 5.8 ± 0.28 with a saturating concentration of approximately 12 nM BMP2 (Fig. 3C). A sub-saturating value of 8 nM BMP2 was chosen for Gremlin 1 assays (Fig. 3C). The IC_50_ value for bacterially-expressed Gremlin 1 is 6.4 ± 0.65 nM, comparable to the IC_50_ value for CS-Gremlin of 6.0 ± 0.33 nM (Fig. 3C; Table 1). Endotoxin levels for Gremlin 1 preparations are approximately 250-fold lower that the LOCE threshold (Table 1). A typical yield of pure Gremlin 1 is approximately 10 mg per L of 2-YT broth culture. For the culture of small intestinal epithelial organoids, 10 mg of Gremlin 1 is sufficient for approximately 30 L of media at a concentration of 25 nM. For the production of Gremlin 1 we estimate a time commitment of two days and material cost of <£10 per litre of organoid media (Table 1). Typically, the cost for one litre of organoid media containing CS-Gremlin 1 at a concentration of 25 nM is >£3,500.

### Use of R-spondin 1 and Gremlin 1 in organoid media formulation

**Figure 4:**
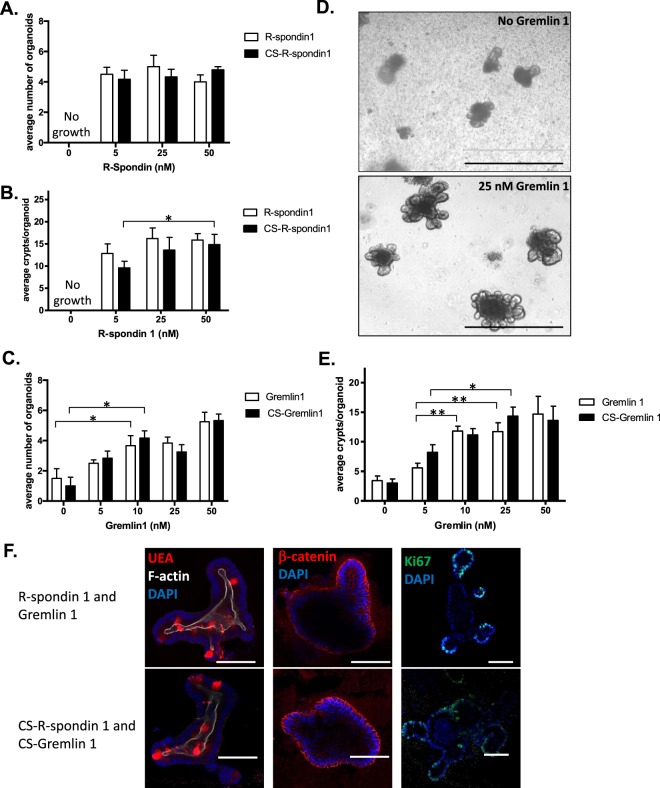
Optimal concentrations of recombinant R-spondin 1 and Gremlin 1 for organoid growth. (**A**) Media formulated with R-spondin 1 or CS-R-spondin 1 equally sustains growth of murine small intestinal epithelial organoids at a minimal concentration of 5 nM. Organoid multiplicity was determined as the average count of organoids that grew under each treatment condition averaged across 8 biological replicates. There were no significant differences in organoid multiplicity between the treatment concentrations. No organoid growth was observed in the absence of R-spondin 1 or CS-R-spondin 1. (**B**) Crypt multiplicity per organoid when grown for 8 days in media supplemented with either R-spondin 1 or CS-R-spondin 1 determined as the average crypt count for 10 organoids with each treatment condition, averaged across 8 biological replicates. There was no significant difference in crypt multiplicity amongst R-spondin 1 treatments. For CS-Rspondin 1, *indicates significant difference in crypt multiplicity between concentrations of 50 nM and 5 nM at p < 0.01, No organoid growth was observed in the absence of R-spondin 1 or CS-R-Spondin 1. (**C**) Gremlin 1- or CS-Gremlin 1-supplemented media equally sustains maximal organoid growth at a minimum concentration of 10 nM. Organoid multiplicity was determined as the average count of organoids that grew under each treatment condition averaged across 8 biological replicates. *Significant difference in organoid multiplicity between 0 nM and 10 nM treatment groups, p < 0.01. Note that Gremlin 1 is not strictly required in growth media as some organoid growth is maintained in its absence. (**D**) Representative examples of organoids cultured in media lacking Gremlin 1 (upper panel) or containing 25 nM Gremlin 1. Scale bar, 200 μm. (**E**) Addition of 10 nM of Gremlin 1 or 25 nM CS-Gremlin 1 is sufficient for maximal crypt multiplicity per organoid after 8 days growth. Average crypt number is the average crypt count for 10 organoids with each treatment condition, averaged across 8 biological replicates. * and ** denote significant differences between 10 nM and 25 nM Gremlin 1 treatment groups and the the 5 nM Gremlin 1 treatment group at p < 0.01 and p < 0.001, respectively. There were no significant differences in crypt multiplicity amongst 10, 25 and 50 nM Gremlin 1 or CS-Gremlin treatment groups. (**F**) Comparison of molecular markers in organoids grown in bacterially-derived (top panels) and commercially-sourced growth factors (lower panels). Left panels – detection of secretory cells and F-actin using Rhodamine-conjugated UEA and Alexa Fluor 647-conjugated Phalloidin. Middle panels – indirect immune-fluorescent detection of β-catenin. Right panels - indirect immune-fluorescent detection of Ki67.

We compared the ability of bacterially-derived R-spondin 1 and Gremlin 1 with the corresponding commercially-sourced growth factors, CS-R-spondin 1 and CS-Gremlin 1 to sustain growth of murine small intestinal epithelial organoids. We found no significant differences equal to or below a threshold value of p < 0.01 for activity measurements comparing R-spondin 1 and CS-R-spondin; both were sufficient, at a minimum concentration of 5 nM, to support the growth of organoid cultures (Fig. 4A). We analysed crypt multiplicity in organoids as a more sensitive probe of R-spondin 1 activity. The addition of bacterially-derived R-spondin 1 or CS-R-spondin 1 to organoid media resulted in a similar number of crypts per organoid after 8 days of growth (Fig. 4B). There was no statistically significant differences in crypt multiplicity amongst the R-spondin 1 treatment groups. Similar results were obtained for CS-R-spondin 1, except that treatment of organoids with 50 nM of the protein led to a significantly higher crypt multiplicity than with 5 nM, but not with 25 nM (Fig. 4B). We routinely use 25 nM R-spondin 1 in organoid culture media.

The addition of Gremlin 1 to organoid media is not strictly required for growth; we find that media formulations that lack Gremlin 1 and other BMP signalling inhibitors (such as Noggin) can sustain growth of murine intestinal epithelial organoids for up to 2–3 passages as previously described^5^. Gremlin 1 or CS-Gremlin 1 have similar activities in organoid media and at 10 nM either protein sustains organoid culture (Fig. 4C). Analysis of crypt multiplicity after 8 days growth indicates that minimum concentrations of 10 nM Gremlin 1 or 25 nM CS-Gremlin 1 yield the maximum number of crypts per organoid (Fig. 4D,E). We routinely use 25 nM of Gremlin 1 in organoid media formulations, sufficient for sustaining growth for at least 20 serial passages.

We then compared the cellular localisation of selected molecular markers within the epithelial monolayer of organoids culture in media containing either bacterially-derived or commercially-sourced R-spondin 1 and Gremlin 1 (Fig. 4F). We did not detect differences in the cellular localisation or distribution of the following molecular probes: UEA, phalloidin, DAPI and antibodies raised against β-catenin or the Ki67 antigen.

### Application of the protocol to organoids from other tissues

**Figure 5:**
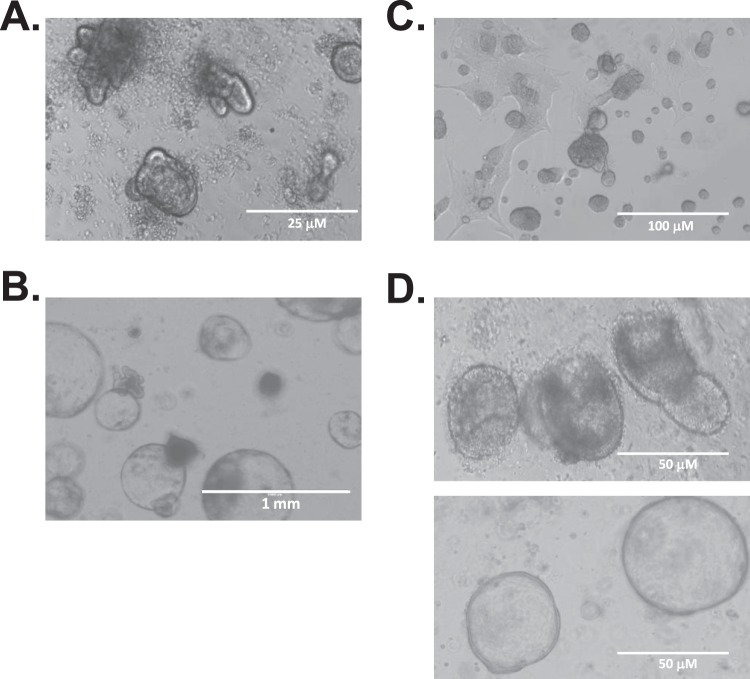
Growth media formulated with R-spondin 1 or Gremlin 1 can sustain growth of various types of organoids. The corresponding media formulations (Table 2) were used for the culture of organoids derived from: (**A**) murine colon epithelia, (**B**) murine APC^min/−^ small intestinal tumours, (**C**) murine mammary gland and (**D**) human biopsy-derived colon epithelia (top panel) and human colon adenocarcinoma (bottom panel).

To test the general applicability of the bacterially-derived growth factors to support organoid growth, we cultured organoids derived from a number of tissues types in corresponding media formulations (Table 2). These included murine tissues including colon epithelia, APC^min/−^ small intestinal tumours and mammary gland (Fig. 5A–C) as well as organoids derived from clinical biopsies of human colon epithelia and patient-matched colon cancer tumours (Fig. 5D).

In all cases except for the human biopsy-derived colon epithelial organoids, the derived cultures were expanded a minimum of four passages and frozen for long-term storage in liquid nitrogen. Organoids were thawed, re-cultured and passaged prior to imaging (Fig. 5A–D). Human colon epithelial organoids were derived from clinical biopsies and passaged 4 times prior to imaging – we find that growth rates and recovery rates after a freeze-thaw cycle for these organoids are low, preventing efficient expansion of the cultures. Media containing bacterially-expressed R-spondin 1 and Gremlin 1 supports the growth of all other types of organoids for greater than 20 serial passages.

## Discussion

Organoid culture, although still being refined for some epithelial tissues, has become a widely-used research tool. Current implementation of organoid technology has graduated from proof-in-principle studies to hypothesis testing in the field of epithelial physiology, stem cell homeostasis, the molecular underpinnings of genetic diseases such as cancer^1^ and cystic fibrosis^6^, recapitulation of epithelial and tumour microenvironments^7^ and host-virus interactions^8^. We report here the production of known activities of Gremlin 1 and R-spondin 1 derived from bacterial expression for use in organoid media. We demonstrate that bacterially-derived R-spondin 1 and Gremlin 1 are comparable to the commercially-sourced growth factors in terms of cellular activities. Importantly, we do not detect differences in organoid growth parameters or more even subtle differences in molecular marker localisation within the organoid epithelia with culture in media formulated with bacterially-derived or commercially-sourced growth factors. We further demonstrate the ability of media formulations containing bacterially-derived R-spondin 1 and Gremlin 1 to sustain the growth of organoids derived from a number of tissue types such as murine colon, mammary gland and APC^min/−^ small intestinal tumours and human colon epithelial and colon adenocarcinoma biopsies.

The production of bacterially-derived R-spondin 1 and Gremlin 1 using our protocols has major advantages over conventional sources of the growth factors derived from conditioned media or commercial sources: (i) the R-spondin 1 and Gremlin 1 preparations are highly pure and devoid of impurities such as serum growth factors that may impact organoid growth; (ii) the use of precise cellular activities of the growth factors in organoid media minimises batch-to-batch variation; and (iii) a substantial reduction in cost of organoid culturing. The protocol for generation of media containing recombinant R-spondin 1 and Gremlin 1 is therefore a leap forward in the ability to generate reproducible growth conditions for both routine organoid culture and large-scale applications such as genetic and chemical screens or clinical tissue biobanking previously reported for limited sample sets of colon cancers^1,2^. We have further demonstrated the application of bacterially-derived growth factors to the culture of organoids derived from a range of tissues including murine small intestine and colon epithelia, mammary gland and human colon epithelia and colon cancer biopsies. We envision that the use of organoid media formulations with defined cellular activities of growth factors will spur the emergence of new applications for organoids that, in particular, require large-scale, cost-effective culture.

One caveat in the implementation of our protocols is the requirement for an animal cell-derived surrogate extracellular matrix. Currently, supports such as Matrigel, produced from Engelbreth-Holm-Swarm mouse sarcoma cells, and may contain serum impurities that impact organoid growth. However, synthetic matrices have emerged in the literature and show substantial promise for supporting organoid growth^9^. Further development of synthetic matrices and their use in conjunction with our protocols will ultimately lead to a fully-defined organoid culture system.

## Materials and Methods

### Expression plasmids

The pR-spondin 1 construct for production of R-spondin 1 is based on a previously reported pETDuet-based vector^10^ for dual expression of a R-spondin 1-containing fusion protein (maltose binding protein, a tetra-cysteine tag, a thrombin cleavage site, residues 21–145 of human R-spondin 1 (Uniprot: O60565) and a hexa-histidine tag) and the *E. coli* disulphide bond isomerase, *DsbC*. We have inserted the coding sequence of the Avi-tag upstream of the R-spondin 1 transgene by digestion of the original construct with *BamH*I and *Not*I and Gibson assembly to create pR-spondin 1 (Fig. 2A).

Plasmids for testing R-spondin 1 activity in the potentiation of Wnt pathway activity are the Super 8x TOPFlash (SuperTop; Addgene 12456) and the *Renilla* Luciferase control reporter vector pRL-SV40P (Promega).

The expression construct, pHAT4-NΔ-Grem1, encoding the hexa-histidine tag, TEV protease cleavage site and amino acids 72–184 of human Gremlin 1 (Uniprot: O60565), has been described previously^4^ (Fig. 3A). Expression of human BMP2 from the bacterial plasmid pBAT-BMP2 and its purification have been detailed elsewhere^4^.

### Cell lines

HEK293T and C2C12 cells were obtained from ATCC. The cell lines are routinely screened for mycoplasma contamination and validated by short tandem repeat DNA profiling (STR) and DNA fingerprinting as described in the ATCC SDO Workgroup ASN-0002 Standards document^11^.

### Organoid media components

Components of the respective media formulations for each organoid type used in the study, listed in Table 2, are as follows: Base media - Advanced DMEM F12 (Gibco) containing 1X penicillin/streptomycin and 10 mM HEPES buffer pH 7.0. Supplements include the following: B27 and N2 supplements (Gibco), N-acetyl-L-cysteine (NAC; Sigma-Aldrich), human gastrin 1 (Sigma-Aldrich), A83-01 (Sigma-Aldrich), SB202190 (Sigma-Aldrich), prostaglandin E2 (PGE2; Sigma-Aldrich), Gastrin I (Sigma-Aldrich), nicotinamide (Sigma-Aldrich) and Primocin (InvivoGen). Commercially-sourced growth factors used in the study are: R-spondin 1 (amino acids 31–263; cat. 4645-RS-025), Gremlin 1 (cat. 5190-GR-050), FGF-2 (cat. 233-FB-010), Neuregulin-1/NRG-1 (cat. 396-HB/CF) and EGF (cat. 236-EG-200), all from R&D Systems. All organoids were grown in growth factor reduced, serum free Matrigel (Fisher Scientific).

### Expression and purification of R-spondin 1

Expression of the R-spondin 1 fusion protein from pR-spondin 1 was carried out in the host *E. coli* strain SHuffle^®^ T7 *E. coli*/K12, following a protocol from the Pioszak laboratory^10^. An overnight starter culture was used to inoculate 2 × 1 L of Luria Broth (LB) media in 2 L baffled Erlenmeyer flasks and grown to an OD_600_ of 0.7 with shaking at 200 rpm. Flasks were transferred to a 16 °C shaker and expression was induced for 16 hours with 0.4 mM isopropyl β-D-1-thiogalactopyranoside (IPTG). Unless otherwise noted, all subsequent steps were carried out at 4 °C. Cells were harvested by centrifugation at 4000 × g for 10 minutes and the pellet was resuspended in 10 ml of resuspension buffer (50 mM Tris-HCl pH 7.5 containing 150 mM NaCl and 10% glycerol, protease inhibitor cocktail; Roche) per 1 L culture. Cells were lysed in an Avestin EmulsiFlex C5 homogeniser and lysates were clarified by centrifugation at 48,000 g for 30 min. The supernatant was transferred to a 50 ml conical tube containing 2 ml of a 50% slurry of Ni^2+^-agarose pre-equilibrated with resuspension buffer and absorption of MBP-R-spondin 1 to Ni^2+^-agarose was carried out for 1 h with gentle agitation. Lysate and Ni^2+^-agarose beads were transferred to a 14 cm Econo-Pac column (Bio-Rad), washed thrice with 10 ml of resuspension buffer containing 25 mM imidazole and MBP-R-spondin 1 was eluted in 1 ml fractions of resuspension buffer containing 265 mM imidazole.

Eluted fractions containing MBP-R-spondin 1 were pooled and concentrated using an Amicon Ultra-15 centrifuge filter (Millipore). Buffer exchange into disulphide reshuffling buffer (50 mM Tris base, pH 8.0 containing 150 mM NaCl, 5% glycerol, 1 mM EDTA, 5 mM reduced glutathione and 1 mM oxidized glutathione) was carried out using a PD10 Sephadex G25 desalting column (GE Healthcare). The concentration of the pooled fractions was diluted to 1 mg/ml with disulphide reshuffling buffer and incubated for 12 hours at room temperature. The reshuffled protein was injected onto a HiLoad Superdex 200 pg 16/600 column (GE Healthcare) equilibrated in resuspension buffer and resolved fractions containing MBP-R-spondin 1 (identified by SDS-PAGE) were pooled and concentrated using an Amicon Ultra-14 centrifuge filter. Buffer exchange was carried out using a PD10 Sephadex G25 desalting column (GE Healthcare) equilibrated in IEX buffer (50 mM Na^2+^/K^+^ phosphate buffer pH 7.0 containing 5% glycerol). At this stage, MBP-R-spondin 1 fusion protein can be analysed to determine concentration (using the Bradford protein assay; Bio-Rad) and used in organoid growth media at a concentration of 25 nM (see Results).

MBP-R-spondin 1 was incubated with 5 U of Thrombin (Sigma) per mg protein for 8 hours at room temperature and the cleaved R-spondin 1 moiety was resolved using cation exchange chromatography (IEX) with a HiTrap SP HP 5 ml column on an AKTA Purifier equilibrated in IEX buffer (15 mM sodium phosphate buffer containing 5% glycerol) with a linear gradient to 1 M NaCl in IEX buffer. R-spondin 1-containing fractions (identified by SDS-PAGE) were pooled, concentrated and buffer exchanged using a PD10 Sephadex column equilibrated in phosphate buffered saline (PBS). The cellular activity of purified R-spondin 1 was determined by Wnt pathway reporter assay (see below).

### Expression and purification of Gremlin 1

Human Gremlin 1 was expressed from a T7-based His-tag vector, His-ΔN-Gremlin 1 (encoding amino acids 72–184 of Gremlin 1), in the *E. coli* strain BL21 (DE3) as follows: cells from glycerol stock were plated on L-agar plates with 100 μg/ml of ampicillin and grown overnight. Cells from the plates were grown in baffled 2 L flasks in 1 L of 2xYT media until an OD_600_ of 0.8 at which point Gremlin 1 expression was induced with 400 μg/ml IPTG for three hours at 37 °C. Cells were harvested by centrifugation for 20 minutes at 4,000 × g and stored frozen at −20 C. The frozen pellet from 1 L of Gremlin 1 expressing culture was resuspended in lysis buffer (50 mM Tris base pH 8.0, 2 mM EDTA pH 8.0, 10 mM DTT, 0.5% Triton-X100) and lysed using an Emulsiflex C5 homogeniser. The lysate was incubated for 20 minutes at 25 °C with DNase I (Sigma) and 4 mM magnesium chloride and clarified by centrifugation for 30 minutes at 15,000 × g. The supernatant was discarded and the pellet fraction containing insoluble inclusion bodies was washed by resuspension in lysis buffer followed by centrifugation for 30 minutes at 15,000 × g. The pellet was re-washed with lysis buffer containing 1 M NaCl and finally with lysis buffer without Triton-X100. The pellet was resuspended in 5 ml of 100 mM Tris(2-carboxyethyl)phosphine (TCEP), to which 15 ml of 50 mM Tris-HCl pH 8.0, 0.5 mM EDTA containing 8 M guanidine hydrochloride (GndHCl) was added (resulting in a final concentration of 6 M GndHCl and 25 mM TCEP). The resuspended pellet was incubated for 15 minutes at 25 °C, clarified by centrifugation (20 min, 15000 × g) and the supernatant diluted to 100 ml with 6 M urea, 20 mM HCl. The solubilised protein solution was then mixed rapidly with 900 ml refolding buffer (50 mM Tris pH 8.0, 1 M 3-[3-(1,1-bisalkyloxyethyl)pyridin-1-yl]propane-1-sulfonate (PPS), 50 mM ethylenediamine, 2 mM EDTA, 2 mM cysteine and 0.2 mM cystine) and left for 7 days at 4 °C to allow the correct formation of the disulphide bonds.

After centrifugation (15,000 × g for 30 mins), the pH of the refolded protein solution was adjusted to 7.0 and loaded onto a 5 ml HiTrap SP HP column equilibrated with binding buffer (20 mM Hepes, pH 7.0). Gremlin 1 was eluted using a linear, 75 ml gradient, of 0% to 100% elution buffer (20 mM Hepes, pH 7.0, 1 M NaCl) and peak fractions, analysed by SDS-PAGE, were pooled. At this stage of the purification, Gremlin 1 can be used in small intestinal epithelial organoid media at a concentration of 25 nM.

Further purification of Gremlin 1 proceeds with the addition of acetonitrile and trifluoroacetic acid to a concentration of 10% and 0.1%, respectively. The sample was loaded onto an ACE 5 C8-300 (4.6 × 250 mm) reversed phase column (HiChrom) equilibrated with binding buffer (10% ACN, 0.1% TFA). Proteins were eluted with a linear 20 ml gradient of 20% to 40% ACN containing 0.1% TFA. Peak fractions with pure protein (analysed by SDS-PAGE) were pooled and their concentration determined using absorbance at 280 nm with a calculated molar absorption coefficient of 11,460 mol^−1^·cm^−1^. Aliquots of 500 μg pure Gremlin 1 were dried under vacuum in a centrifugal concentrator and stored at −80 °C. The protein is stable for at least 24 months when stored in this way. Prior to use, purified Gremlin 1 was reconstituted in PBS at a concentration of 1 mg/ml and cellular activity determined by alkaline phosphatase assay (see below).

### Cellular reporter assay of R-spondin 1 activity

The cellular activities of MBP-R-spondin and R-spondin 1 fractions were determined by their ability to potentiate Wnt3A-induced pathway activity in HEK293T cells, designated as WPC50 (Wnt pathway activity potentiation constant), and defined as the concentration of added R-spondins sufficient for 50% of total reporter activity. Cells were plated onto 24-well tissue culture plates (75,000 cells per well) in DMEM media supplemented with 10% fetal calf serum (Gibco) and transfected with 100 ng SuperTop and 10 ng pRL-SV40P plasmids. Transfected cells were treated with Wnt3A-conditioned media (WCM; produced from Wnt3A-expressing L-cells; ATCC CRL-2646) diluted 1:4 with growth media and varying concentrations of R-spondin 1 (typically 1–150 nM). At 16 hours post-treatment, cells were harvested and reporter activities determined with the Promega Stop & Glo kit using a PHERAstar microplate reader. Normalised luciferase activity was determined by dividing firefly luciferase values by the *Renilla* luciferase values. SuperTop activity values for each concentration of R-spondin were the average of four individual biological replicates and reported with standard deviation (SD). All WPC50 values were calculated using GraphPad Prism Software Version 5 by fitting the Hill equation to the dose-response data.

### Cellular Gremlin 1 activity

The cellular activity of purified Gremlin 1 is defined as the IC_50_ for inhibition of BMP2-induced cellular activity using the alkaline phosphatase assay (ALP assay)^12^. Optimal concentration of BMP2 for use in Gremlin 1 activity assays was determined as follows: C2C12 cells (low passage number is critical) were plated into 96-well plates and cultured in DMEM containing 10% FBS. A serial dilution of BMP2 (0 nM–15 nM) was added to the cells and incubated for 48 hrs. Cells were washed with PBS and lysed in 0.56 mM 2-amino-2-methyl-propan-1-ol and 1.0% SDS, pH 10.0. ALP activity was measured using the substrate *p*-nitrophenyl phosphate (Sigmafast pNPP pellets; Sigma Aldrich) and absorbance read at 405 nm using a PHERAstar FS plate reader. The EC_50_ value for BMP2-induced ALP activity was determined as 5.8 ± 0.28 nM with saturating activity at approximately 12 nM. Activity values for each concentration of BMP2 were the average of 3 individual experimental replicates reported with SD. The sub-maximal value of 8 nM BMP2 was chosen for use in Gremlin 1 activity assays.

Determination of the Gremlin 1 IC_50_ for inhibition of BMP2-induced ALP activity was carried out as above, using serial dilutions of Gremlin 1 (1–25 nM) in the presence of 8 nM BMP2. Activity values for each concentration of Gremlin 1 were the average of 4 individual experimental replicates reported with SD. All kinetic parameters for ALP assays were calculated using GraphPad Prism Software Version 5 by fitting the Hill equation to the dose-response data.

### Endotoxin assay

Levels of endotoxin in the R-spondin 1 and Gremlin 1 preparations was determined using the ToxinSensor system (GenScript) according to manufacturer’s protocol. Values were determined from at least two individual preparations of R-spondin 1 and Gremlin 1 fractions, measured with two technical replicates and reported as average values with SD. We have set a maximum endotoxin concentration of 0.5 EU/ml in organoid media, below the Lowest Observed Cellular Effect (LOCE) threshold for detectable cellular cytokine production and NF-kB activation, previously determined in cell culture assays^13^.

### Derivation of organoid cultures

Murine small intestinal epithelial organoids and colon epithelial organoids (murine and human) were derived according to Sato *et al*.^14^ and Sato *et al*.^15^, respectively. Murine mammary gland organoids were derived as in Nguyen-Ngoc *et al*.^16^. A summary of all media formulations used in the study is shown in Table 2. All derived organoid cultures were grown in 48-well CellStar plates (Sigma-Aldrich), embedded in a 25 μL droplet of Matrigel (Corning), cultured in 200 μL of the corresponding organoid media in an incubator at 37 °C and 5% CO_2_.

All murine tissue used in the study was collected from euthanized mice by trained technicians within the Wellcome Trust Gurdon Institute facility, following UK Home Office approved guidelines. Human colon epithelial- and colon cancer-derived organoids were from the Addenbrooke’s Hospital Tissue Biobank donated under informed, explicit, patient consent. Human tissue obtained for the study was under generic Biobank ethical approval and all applicable institutional guidelines and regulations regarding the use of human tissue were followed.

### Optimisation of R-spondin 1 and Gremlin 1 concentration in media formulations

Optimisation of R-spondin 1 and Gremlin 1 concentrations for use in media for culture of murine small intestinal epithelial organoids were determined by growing organoids in individual wells with variable concentrations of R-spondin 1 (0–100 nM) or Gremlin 1 (0–25 nM) in otherwise fully-supplemented organoid growth media. Growth characteristics (multiplicity and crypt number) of organoids were tested 8 days post-plating. Organoid multiplicity was determined as the average organoid count for each treatment from 8 different wells, reported with SD. Organoid crypt number was determined as the average number of crypts from 10 organoids in individual wells, averaged across 8 wells, reported with SD. Statistical differences in organoid multiplicity and crypt number amongst the individual concentrations and growth media conditions were determined by two-tailed T-test, calculated using Excel software.

### Fluorescent detection of molecular markers in organoids

Organoids were seeded and cultured in eight-chamber μ-slides (Thistle Scientific) for three days and fixed in 4% formaldehyde solution and molecular markers were visualised following the protocol from Fatehullah^17^. Molecular probes used for fluorescent detection and indirect immunofluorescence in organoids were as follows: Rhodamine-conjugated Ulex europeaus agglutinin 1 (UEA; Vector Laboratories), Alexa Fluor 647 Phalloidin (Thermo Fisher Scientific), and antibodies raised against β-catenin (BD Transduction Laboratories) or Ki67 (Thermo Fisher Scientific). The secondary antibodies were Goat Anti-Mouse IgG Alexa Fluor 555 and Goat Anti-Rabbit IgG Alexa Fluor 488 (both from BD Transduction Laboratories), respectively. Slides were mounted using DAPI Fluoromount G (Southern Biotech).

Fluorescent images were taken by a Zeiss LSM 700 laser scanning confocal microscope using an oil-immersion 40x objective. Images were processed using Zen and ImageJ software.

## Supplementary information

Supplementary Figures

## Data Availability

All data generated throughout the study is available from the corresponding authors.

## References

[1] van de Wetering M (2015). Prospective derivation of a living organoid biobank of colorectal cancer patients. Cell.

[2] Fujii M (2016). A Colorectal Tumor Organoid Library Demonstrates Progressive Loss of Niche Factor Requirements during Tumorigenesis. Cell stem cell.

[3] Moad HE, Pioszak AA (2013). Selective CGRP and adrenomedullin peptide binding by tethered RAMP-calcitonin receptor-like receptor extracellular domain fusion proteins. Protein science: a publication of the Protein Society.

[4] Kisonaite M, Wang X, Hyvonen M (2016). Structure of Gremlin-1 and analysis of its interaction with BMP-2. The Biochemical journal.

[5] Sato T (2009). Single Lgr5 stem cells build crypt-villus structures *in vitro* without a mesenchymal niche. Nature.

[6] Dekkers JF (2013). A functional CFTR assay using primary cystic fibrosis intestinal organoids. Nature medicine.

[7] Baker Kristi (2018). Organoids Provide an Important Window on Inflammation in Cancer. Cancers.

[8] Hui Kenrie P Y, Ching Rachel H H, Chan Stan K H, Nicholls John M, Sachs Norman, Clevers Hans, Peiris J S Malik, Chan Michael C W (2018). Tropism, replication competence, and innate immune responses of influenza virus: an analysis of human airway organoids and ex-vivo bronchus cultures. The Lancet Respiratory Medicine.

[9] Gjorevski N (2016). Designer matrices for intestinal stem cell and organoid culture. Nature.

[10] Moad HE, Pioszak AA (2013). Reconstitution of R-spondin:LGR4:ZNRF3 adult stem cell growth factor signaling complexes with recombinant proteins produced in Escherichia coli. Biochemistry.

[11] International Cell Line Authentication Committee (ICLAC). Guide to Human Cell Line Authentication, https://webstore.ansi.org/RecordDetail.aspx?sku=ANSI%2fATCC+ASN-0002-2011.

[12] Katagiri T (1994). Bone morphogenetic protein-2 converts the differentiation pathway of C2C12 myoblasts into the osteoblast lineage. The Journal of cell biology.

[13] Schwarz H, Schmittner M, Duschl A, Horejs-Hoeck J (2014). Residual endotoxin contaminations in recombinant proteins are sufficient to activate human CD1c + dendritic cells. PloS one.

[14] Sato T, Clevers H (2013). Primary mouse small intestinal epithelial cell cultures. Methods in molecular biology.

[15] Sato T (2011). Long-term expansion of epithelial organoids from human colon, adenoma, adenocarcinoma, and Barrett’s epithelium. Gastroenterology.

[16] Nguyen-Ngoc KV (2015). 3D culture assays of murine mammary branching morphogenesis and epithelial invasion. Methods in molecular biology.

[17] Fatehullah A, Appleton PL, Nathke IS (2013). Cell and tissue polarity in the intestinal tract during tumourigenesis: cells still know the right way up, but tissue organization is lost. Philosophical transactions of the Royal Society of London. Series B, Biological sciences.

