# Author Correction: Organoid culture media formulated with growth factors of defined cellular activity

**DOI:** 10.1038/s41598-020-68552-8

**Published:** 2020-07-09

**Authors:** Manuela Urbischek, Helena Rannikmae, Thomas Foets, Katharina Ravn, Marko Hyvönen, Marc de la Roche

**Affiliations:** Department of Biochemistry, University of Cambridge, Cambridge, UK

Correction to: *Scientific Reports* 10.1038/s41598-019-42604-0, published online 17 April 2019.

The Article contains errors in Table 2, where the units used for components ‘EGF’ and ‘FGF’,

“μg/m”

should read:

“ng/ml”

Additionally, the units used for component ‘NRG-1′,

“ng.ml”

should read:

“ng/ml”

In the same table, the ‘Human colon epithelia’ entry for WCM,

“—”

should read:

“0.5×”

In addition, the ‘Human colon carcinoma’ entry for WCM,

“0.5×”

should read:

“—”

