# Supplementary material for: Organoid culture media formulated with growth factors of defined cellular activity
Source: Sci Rep. 2019 Apr 17;9:6193. doi: 10.1038/s41598-019-42604-0 (PMC6470207; doi:10.1038/s41598-019-42604-0)

SUPPLEMENTAL FIGURES. Organoid culture media formulated with growth factors of defined cellular activity.

Manuela Urbischek, Helena Rannikmae, Thomas Foets, Katharina Ravn, Marko Hyvönen and Marc de la Roche

Supplemental Figure 1. Elution of MBR-R-spondin 1 from the SEC column. NiNTA-purified MBP-R-spondin 1 was subject to a disulphide shuffling step, concentrated to 15 ml and applied to a 200 pg 16/600 SEC column. MBP-R-spondin 1 eluted in two peaks, measured by UV absorbance of 280 nM – a peak centred at 150 ml elution volume corresponding to high molecular weight aggregates and a peak centred on 210 ml elution volume corresponding to active MBP-R-spondin 1.

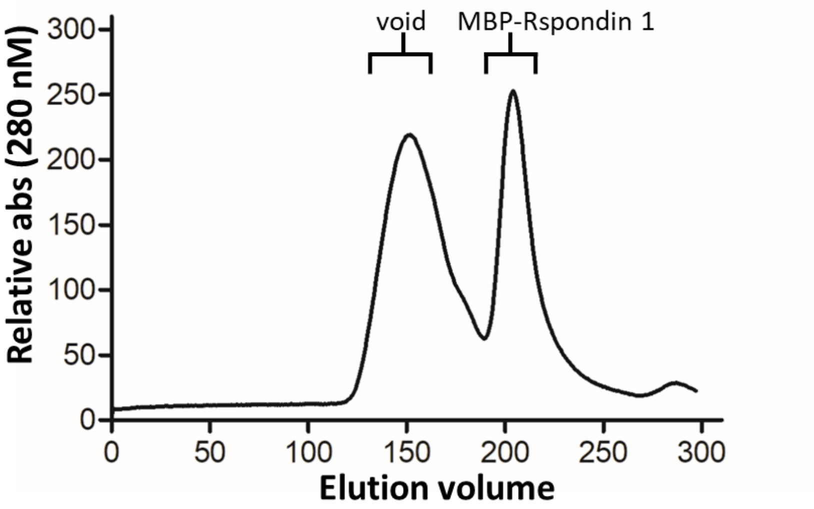

Supplemental Figure 2. Full SDS-PAGE gel of purified fractions from the preparation of R-spondin 1 and Gremlin 1.

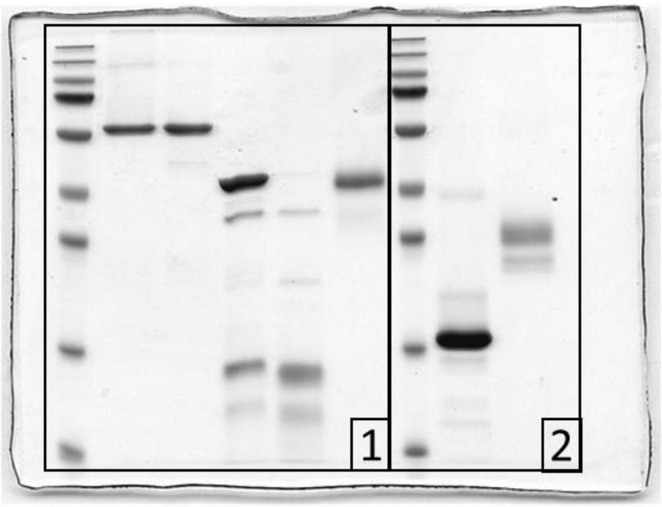

Supplement: Supplementary file 1 — Supplementary Figures [file 41598_2019_42604_MOESM1_ESM.pdf]
